# Supplementary material for: Membrane curvature sensing of the lipid-anchored K-Ras small GTPase
Source: Life Sci Alliance. 2019 Jul 11;2(4):e201900343. doi: 10.26508/lsa.201900343 (PMC6625090; doi:10.26508/lsa.201900343)
Supplement: Supplementary file 1 [file LSA-2019-00343_TableS1.docx]

|  | Curvature directions | Curvature magnitudes | K-Ras | tK | H-Ras | tH |
| --- | --- | --- | --- | --- | --- | --- |
| Nanobars | Positive |  | Low |  |  | High |
| BAR_amph2_ | Positive |  | Low | Low | High | High |
| BAR_FCC_ | Positive |  | Low |  |  | High |
| BAR_IRS53p_ | Negative |  | No change |  |  | Low |
| BAR_FCH_ | None |  | No change |  |  | No change |
| Hypo | Fluctuations |  | High | High | Low | Low |
| GPMV ΔOsm  (Hypertonic and low hypotonic 🡪 bending) | Fluctuations |  |  | High |  | Low |
| GPMV ΔOsm  (High Hypotonic 🡪 membrane tension) | Fluctuations |  |  | Low |  | Low |
| Vesicle size (small 🡪 large diameters) | Positive |  | High |  |  |  |

**Table 1.** Membrane curvature-induced changes in Ras spatiotemporal organization on biomembranes and synthetic bilayers.
